# Capability well-being in mothers and fathers of autistic children: a cross-sectional study from China

**DOI:** 10.1186/s40359-025-03507-0

**Published:** 2025-10-30

**Authors:** Huanyu Zhang, Shanquan Chen, Jiazhou Yu, Fei Chen, Jinting Yan, Li Wang

**Affiliations:** 1 Clinical Big Data Research Center, The Seventh Affiliated Hospital, Sun Yat-sen University, Shenzhen, 518107 China; 2 School of Public Health, The University of Hong Kong, Hong Kong SAR, China; 3 Jockey Club School of Public Health and Primary Care, The Chinese University of Hong Kong, Hong Kong SAR, China; 4 School of Foreign Languages, Hunan University, Changsha, 410082 China; 5 Cangzhou Research Centre for Child Language Rehabilitation, Cangzhou Normal University, Hebei, 061001 China; 6 Shenzhen Research Institute, The Chinese University of Hong Kong, Shenzhen, 518000 China; 7 Department of Educational Psychology, The Chinese University of Hong Kong, Shatin, N.T., Hong Kong SAR, China

**Keywords:** Autism, Parental well-being, Gender disparity, Predictors

## Abstract

**Background:**

Parents of autistic children often experience reduced well-being, with most studies focusing on negative outcomes. However, fewer studies have applied capability approach to assess parental abilities to achieve positive functioning. This study aims to evaluate well-being in parents of autistic children, with a particular attention to differences between mothers and fathers.

**Methods:**

We conducted a cross-sectional study of 366 parents of children aged 1–17 years with a definitive diagnosis in China, including 318 mothers and 48 fathers. Parental well-being was assessed using the Investigating Choice Experiments Capability Measures for Adults (ICECAP-A), which captures key domains of well-being. We employed the United Kingdom tariffs to calculate index scores of the ICECAP-A. Stepwise multivariate linear regression models were performed to identify predictors of parental well-being.

**Results:**

The mean score of the ICECAP-A among parents was 0.672 (SD 0.214), with a significant gender disparity (*p* = 0.012, rank-biserial correction = 0.131, 95%CI [0.030, 0.227]). Mothers perceived a lower level of well-being than fathers in the domains of autonomy (β=-0.022, *p* < 0.01) and achievement (β=-0.021, *p* < 0.001). Risk factors for impaired parental well-being included more severe autistic symptoms, lower socioeconomic status of parents, and a considerably longer time spent on caretaking of the child.

**Conclusions:**

Professionals should prioritize gender-specific intervention programs tailored to the distinct needs of mothers and fathers, and actively promote and facilitate father involvement in the childcare. Evidence-based psychosocial support services need to be specifically designed for high-risk parent populations, especially those facing intensive caregiving burdens, socioeconomic disadvantages, or parenting autistic children with more severe symptoms.

## Introduction

Autism spectrum disorder (ASD, hereafter termed autism) is a group of neurodevelopmental conditions characterized by deficits in social interaction and communication, as well as the presence of restrictive, repetitive, and ritualistic patterns of behavior, interests, and activities [1]. The average prevalence estimates of autism ranged from 1.0% to 2.8% across Asia, Europe, and North America [2, 3]. Autistic children often present with a range of behavioral and emotional difficulties that can place substantial mental, emotional, and financial strain on their parents [4, 5]. In China, these challenges are compounded by limited access to diagnostic and intervention services [6] and by cultural stigma, which often associates a child’s disability with parental failure, leading to social isolation, self-blame, and heightened caregiving stress [7–10].

Traditional gender roles place even greater pressure on mothers, who are expected to manage daily care and actively support their children’s development, often at the expense of their own well-being [11, 12]. These gendered caregiving dynamics may lead to different psychological and social outcomes for mothers and fathers [13, 14]. Therefore, examining differences between mothers and fathers in well-being and related factors is essential to understanding how caregiving affects each group and to developing targeted supports. Historically, research on parenting autistic children has primarily focused on mothers, as studies have largely relied on maternal samples [5, 15]. This bias stems from the fact that mothers are more frequently the primary caregivers, leaving fathers’ well-being understudied. Even when fathers were included in research, gender differences were often overlooked in analyses, limiting insights into family-wide needs and functioning [3, 16]. While father-inclusive autism research has gradually increased in the past two decades [17], findings on parental well-being remain inconsistent. Some studies report higher stress, depression, and anxiety in mothers compared to fathers, while others find no significant differences [5, 15]. Further research is needed to clarify these disparities and better understand how parenting autistic children affects mothers and fathers differently.

Well-being refers to an individual’s emotional response to what their life is like [18]. It generally considers physical (feeling full of energy), mental (a sense of optimism), and social (receiving support from family and friends) aspects of a person’s life [19]. Existing research indicates that parents of autistic children often experience negative impacts on their well-being, leading to physical and mental health issues and social isolation [20–22]. However, much of the previous literature has focused on the negative aspects of caregiving, such as stress and health-related problems (e.g., depression, anxiety) [22, 23]. It is important to note that parents with mental health challenges or negative feelings should not be assumed to be unhappy or unfulfilled. Previous studies have also highlighted positive aspects of parenting, such as personal growth and enrichment resulting from raising autistic children [24, 25]. In this context, parenting sense of competence, which refers to parents’ confidence and satisfaction in their caregiving role, has emerged as an important factor influencing parental well-being and may help explain variations in caregiving experiences, both within individuals and between genders [26]. Therefore, a more comprehensive approach should be employed to assess parental well-being concerning positive contributions of parenting experience.

Capability well-being, as defined by the capability approach [27], offers an alternative framework to traditional measures of well-being. This approach emphasizes individuals’ freedom to achieve valuable life activities, focusing on capabilities—what an individual can do and be—rather than mere functioning, or what they actually do [28, 29]. In this paper, we use the term capability well-being to describe this broader view of well-being. The Investigating Choice Experiments Capability Measure for Adults (ICECAP-A) instrument operationalizes capability well-being through five positive domains: stability, attachment, autonomy, achievement, and enjoyment [30]. Unlike traditional well-being measures that assess emotional states (e.g., happiness, distress) or role satisfaction, the capability approach focuses on what individuals are free to do and be. This distinction is important in parenting contexts, where individuals may experience psychological strain but still value and retain capabilities such as maintaining meaningful relationships (attachment), making independent choices (autonomy), or achieving personal goals (achievement). By focusing on capabilities rather than experienced symptoms or functioning alone, the ICECAP-A captures the latent potential and freedoms that shape individuals’ quality of life—offering a complementary, and in some cases, more inclusive framework for understanding well-being.

The ICECAP-A has been widely applied in economic evaluations to capture aspects of well-being beyond health [29, 31]. Recently, our research team has conducted a study to examine the association between adherence to behavioral intervention and capability well-being among parents of autistic children [32]. However, few studies have applied the capability approach to assess positive aspects of well-being beyond health in both mothers and fathers of autistic children. Hence, in this study, we aim to (1) assess levels of capability well-being among Chinese parents of autistic children using the ICECAP-A across five positive domains, (2) identify factors associated with parental capability well-being, and (3) examine differences between mothers and fathers in capability well-being, parenting competence, and key demographic and caregiving characteristics.

## Methods

### Study design and participants

A cross-sectional study was performed in China from March through September, 2023. Since there is lack of a complete sampling frame affected by autism in China, a non-probability convenience sampling method was employed to recruit participants in this study through social media, non-profit agencies, public and private intervention institutions, and parent associations. An online survey was distributed to reach families affected by autism as widely as possible across diverse provinces in China. Inclusion criteria of eligible participants in this investigation included (1) parents or caregivers of autistic children; (2) parenting an autistic child aged 1–17 years at the study entry; (3) a confirmed diagnosis of autism among children. Of the 489 individuals who completed the survey, 385 met all inclusion criteria and provided valid responses. For the purposes of this study, we further restricted the sample to 366 respondents who identified as either the mother or father of an autistic child. While this approach enabled wide geographic reach, it may have introduced sampling bias, as parents more engaged with autism-related services or networks may have been more likely to participate.

Recruitment was carried out using multiple channels to reach caregivers of autistic children across various provinces in Mainland China. Participants were recruited through social media platforms (e.g., WeChat), autism-related parent associations, non-profit organizations, and public or private intervention institutions. Digital flyers and social media posts containing a brief study description and a QR code linking to the online survey were disseminated through these channels. Upon scanning the QR code, potential participants were directed to a landing page on Wenjuanxing (a widely used Chinese online survey platform) where they first reviewed detailed information about the study’s purpose, procedures, and ethical safeguards. They then indicated informed consent by clicking a “Consent to Participate” button before accessing the questionnaire. Participants were clearly informed that their involvement was voluntary and that they could withdraw at any time. No identifying personal information was collected, and responses were fully anonymized. No financial incentives were offered for participation.

This study is part of a broader investigation on Chinese families affected by autism which has received ethical approval from the Research Ethics Committee of the Shenzhen Research Institute, The Chinese University of Hong Kong (PJ-202210B). Informed consent was obtained from all the participants included in this investigation.

### Study measures

#### Capability well-being

Parental capability well-being was measured using the validated Chinese version of the ICECAP-A, which consists of the following five domains: (1) Stability: to feel settled and secure; (2) Attachment: to have love, friendship, and support; (3) Autonomy: to be independent; (4) Achievement: to achieve and progress; (5) Enjoyment: to experience enjoyment and pleasure. Participants rated each attribute on a scale from 1 to 4, with 1 indicating full capability and 4 indicating no capability. Due to the absence of preference-based data in the Chinese population, a value set based on the general population preferences in the United Kingdom was used to calculate ICECAP-A index scores for each domain [33]. The total index score ranged from 0 to 1, with higher scores indicating better capability well-being. The Chinese version of the ICECAP-A demonstrated an acceptable internal consistency (Cronbach’s Alpha = 0.80 in prior research) [34], and in this study, the internal consistency was 0.86.

#### Parent sense of competence

Since parenting sense of competence is an important predictor of parental well-being among parents of autistic children [26], we utilized the validated Chinese version of the Parenting Sense of Competence Scale (PSOC) to assess perception of the participants on their abilities to manage the demands of parenting [35]. The PSOC consists of 17 items divided into two subscales: (1) Efficacy (8 items): reflecting parents’ perceived competence in the parenting role; (2) Satisfaction (9 items): measuring parents’ satisfaction and comfort with the parenting role. Participants rated each item on a 6-point Likert scale ranging from strongly disagree to strongly agree. The total PSOC score ranged from 17 to 102, with higher scores indicating a higher sense of competence. The Chinese version of the PSOC has demonstrated good internal consistency (Cronbach’s Alpha = 0.85 in prior research) [35], and in this study, the internal consistency was 0.80.

#### Level of autism severity

The level of autism severity was assessed by the Chinese version of Clancy Autism Behavior Scale (CABS) in this study [36]. Parents rated 14 behavioral items on three frequency levels: 0 = never; 1 = occasionally; 2 = often. The total score was the sum of the 14 items, with higher scores indicating more severe autism symptoms. The CABS has demonstrated high agreement with diagnostic criteria and good validity [36]. In this study, the internal consistency (Cronbach’s Alpha) was 0.81.

#### Demographic and socioeconomic characteristics

Child characteristics included age, sex, level of severity, currently receiving any intervention (yes vs. no), and school attendance (yes vs. no). Parent and family-level demographic and socioeconomic factors included parent age, marital status (married, divorced/separated, and other), education level (less than college, college, bachelor’s degree, and advanced degree), time on caretaking of the child per day (< 3 h, 3–6 h, 7–12 h, and > 12 h), number of children (1 child vs. >1 child), and annual household income in the past 12 months (measured in 100,000 RMB; 1 RMB = 0.14 USD).

### Statistical analyses

To compare characteristics and well-being between fathers and mothers, descriptive analyses were conducted by mothers and fathers of autistic children. Continuous variables were reported as mean (standard deviation, SD) and tested by Wilcoxon rank-sum tests due to non-normal distributions. Categorical variables were reported as number (percentage) and tested by Fisher’s Exact test where low expected cell frequencies precluded chi-square tests. The effect size for the difference in capability well-being between mothers and fathers was reported using the rank-biserial correlation.

To further explore the predictors of parental capability well-being, we fitted a series of linear regression models with the total index score and five domain scores of the ICECAP-A as outcomes, respectively. A full linear regression model was firstly conducted with all the potential factors, including parental role (mothers vs. fathers), PSOC score, child characteristics (age, sex, level of autism severity, intervention status, and school attendance), and other parent and family variables (age, marital status, education level, time on caretaking of the child per day, number of children, household income). This was followed by a backward stepwise selection process based on the Akaike Information Criterion scores to refine the variables included in the final model. The coefficient and its 95% confidence interval levels (CIs) were subsequently extracted from the final model. Different predictors were retained across the outcome models, as the final set of variables selected depended on the specific outcome being examined. The variance inflation factor (VIF) for each variable included in the final model was assessed to avoid multicollinearity. The results showed that there was no value of VIF greater than 5, indicating the absence of multicollinearity in all the final models.

All the statistical analyses were completed in R version 4.2.2. A *p* value < 0.5 was considered statistically significant.

## Results

Among the 366 eligible participants, 48 (13.1%) fathers and 318 (86.9%) mothers of autistic children were included in this study, with a mean age of 35.5 (SD 6.4). Most (87.7%) of the parents were married, and nearly half (49.5%) of the involved families raised more than one child. For the autistic children, 79.8% of them were males, with a mean age of 5.8 (SD 2.8). The majority (82.8%) of the involved children were receiving intervention at the study entry, with a mean CABS score of 15.3 (SD 5.0). More detailed characteristics of the study sample at child, parent, and family levels are presented in Table 1.

**Table 1:** Descriptive statistics in mothers and fathers of autistic children

	Total *N* = 366	Fathers *N* = 48	Mothers *N* = 318	*p*-value
*Child characteristics*				
Child Age	5.8(2.8)	6.2(3.0)	5.7(2.8)	0.231
Child Sex				0.570
Male	292(79.8%)	40(83.3%)	252(79.2%)	
Female	74(20.2%)	8(16.7%)	66(20.8%)	
Level of autism severity in CABS score	15.3(5.0)	16.9(4.8)	15.0(5.0)	**0.028**
Currently receiving any intervention				0.064
No	63(17.2%)	13(27.1%)	50(15.7%)	
Yes	303(82.8%)	35(72.9%)	268(84.3%)	
School attendance				0.867
No	110(30.1%)	15(31.3%)	95(29.9%)	
Yes	256(69.9%)	33(68.8%)	223(70.1%)	
*Parent and family characteristics*				
Parent Age	35.5(6.4)	36.1(9.3)	35.4(5.9)	0.222
Parent sense of competence score	56.5(8.3)	58.6(7.5)	56.2(8.3)	0.056
Efficacy subscale	31.2(6.6)	33.3(8.2)	30.8(6.2)	**0.029**
Satisfaction subscale	25.3(6.5)	25.2(8.7)	25.4(6.1)	0.757
Marital status				0.636
Married	321(87.7%)	41(85.4%)	280(88.1%)	
Divorced/Separated	33(9.0%)	6(12.5%)	27(8.5%)	
Other	12(3.3%)	1(2.1%)	11(3.5%)	
Education level				0.355
Less than college	82(22.4%)	12(25.0%)	70(22.0%)	
College	76(20.8%)	9(18.8%)	67(21.1%)	
Bachelor’s degree	157(42.9%)	24(50.0%)	133(41.8%)	
Advanced degree	51(13.9%)	3(6.3%)	48(15.1%)	
Time on caretaking of the child per day				**0.001**
< 3 h	89(24.3%)	23(47.9%)	66(20.8%)	
3–6 h	119(32.5%)	9(18.8%)	70(22.0%)	
7–12 h	79(21.6%)	8(16.7%)	71(22.3%)	
> 12 h	79(21.6%)	8(16.7%)	111(34.9%)	
Number of children				0.878
1 child	185(50.5%)	25(52.1%)	160(50.3%)	
> 1 child	181(49.5%)	23(47.9%)	158(49.7%)	
Household income in 100,000 RMB	2.6(5.6)	2.0(2.3)	2.6(5.9)	0.835
*Capability well-being*				
ICECAP index score	0.672(0.214)	0.745(0.178)	0.660(0.217)	**0.012**
Stability	0.142(0.068)	0.157(0.059)	0.140(0.069)	0.106
Attachment	0.151(0.060)	0.156(0.060)	0.150(0.060)	0.469
Autonomy	0.135(0.049)	0.156(0.040)	0.132(0.049)	**< 0.001**
Achievement	0.116(0.044)	0.137(0.039)	0.113(0.044)	**< 0.001**
Enjoyment	0.127(0.049)	0.138(0.044)	0.125(0.049)	**0.035**

Bold values indicate statistical significance at *p* < 0.05

### Aim 1: levels of capability well-being among parents

The mean overall score of the ICECAP-A among parents was 0.672 (SD 0.214), with a significant gender disparity (*p* = 0.012, rank-biserial correction = 0.131, 95%CI [0.030, 0.227]). This score is lower than the Chinese general population score of 0.848 [34]. Specifically, no significant difference was found in the domains of stability (*p* = 0.106) and attachment (*p* = 0.469) between fathers and mothers, while a higher paternal score was reported in the domains of autonomy (*p* < 0.001, *r* = 0.196, 95%CI [0.094, 0.290]), achievement (*p* < 0.001, rank-biserial correction = 0.193, 95%CI [0.093, 0.296]), and enjoyment (*p* = 0.035, rank-biserial correction = 0.110, 95%CI [0.005, 0.221]) compared to the corresponding maternal scores. The effect sizes for these gender differences were small, indicating that while statistically significant, the practical difference in capability well-being between fathers and mothers is modest.

### Aim 2: factors associated with parental capability well-being

In Table 2, we employed the overall and five domain scores of capability well-being as outcomes and identified predictors of well-being, respectively. After controlling other confounding factors, mothers perceived a worse well-being than fathers in autonomy (β=−0.022) and achievement (β=−0.021). Parent sense of competence was positively associated with the overall and all the five domain scores of well-being (*p* < 0.001). Compared to the education level of lower than college, parents with a bachelor’s degree reported a higher score in the overall well-being (β = 0.068) and domains of attachment (β = 0.021), autonomy (β = 0.016), and achievement (β = 0.012). Families with a higher household income reported a higher score in stability (β = 0.001). Parents of children who were receiving intervention reported a higher score in attachment (β = 0.017) than those who were not.

**Table 2:** Predictors of the overall and five domain scores of capability well-being

	ICECAP	Stability	Attachment	Autonomy	Achievement	Enjoyment
	*Beta-coefficient [95% CI]*					
Child age	-	−0.002[−0.004,0.000]	-	-	-	-
Level of severity in CABS score	**−0.005[−0.009**,−0.001]**	−0.001[−0.002,0.000]	**−0.001[−0.002**,−0.000]*	−0.001[−0.002,0.000]	-	**−0.001[−0.002**,−0.000]*
Currently receiving intervention (= Yes)	-	-	**0.017[0.002**,0.032]*	-	0.009[−0.001,0.020]	-
Parent(= Mother)	−0.054[−0.109,0.001]	-	-	**−0.022[−0.035**,−0.008]**	**−0.021[−0.033**,−0.009]***	-
Parent age	**−0.003[−0.006**,−0.000]*	-	-	−0.001[−0.001,0.000]	-	−0.001[−0.001,0.000]
Parent sense of competence score	**0.013[0.010**,0.015]***	**0.004[0.003**,0.005]***	**0.002[0.002**,0.003]***	**0.002[0.001**,0.003]***	**0.002[0.002**,0.003]***	**0.002[0.002**,0.003]***
Marital (Ref = Married)	-	-	-		-	-
Divorced/separated				−0.014[−0.030,0.002]		
Other				−0.016[−0.042,0.009]		
Education level (Ref = Less than college)		-				-
College	0.031[−0.024,0.086]		**0.017[0.000**,0.035]*	0.004[−0.010,0.018]	0.002[−0.010,0.014]	
Bachelor’s degree	**0.068[0.020**,0.116]**		**0.021[0.006**,0.036]**	**0.016[0.004**,0.028]**	**0.012[0.002**,0.022]*	
Advanced degree	0.065[−0.001,0.132]		**0.028[0.008**,0.047]**	0.011[−0.004,0.027]	**0.023[0.009**,0.036]**	
Time on caretaking per day (Ref = < 3 h)				-	-	
3–6 h	−0.019[−0.072,0.034]	0.000[−0.018,0.018]	−0.008[−0.025,0.009]			−0.007[−0.020,0.006]
7–12 h	−0.015[−0.069,0.039]	−0.006[−0.023,0.012]	−0.008[−0.025,0.009]			−0.007[−0.019,0.006]
> 12 h	**−0.074[−0.125**,−0.024]**	**−0.029[−0.045**,−0.014]***	**−0.019[−0.035**,−0.004]*			**−0.025[−0.036**,−0.013]***
Household income	0.003[−0.001,0.006]	**0.001[0.000**,0.002]*	-	-	-	0.001[−0.000,0.001]

Data are presented as coefficients and its 95% confidence intervals. All the factors are listed in the regression analyses initially, followed by a stepwise selection process based on the Akaike Information Criterion (AIC) scores to refine the variables included in the final models. Bold values indicate statistical significance

*p < 0.05

**p < 0.01

***p < 0.001

“-” indicates that the variable was removed in the stepwise selection process

Regarding risk factors that inversely associated with parental well-being, a more severe level of autistic symptoms significantly predicted a lower level of scores in the overall well-being (β=−0.005) and domains of attachment (β=−0.001) and enjoyment (β=−0.001). In contrast to parents who spent fewer than 3 h on caretaking of the child per day, parents who spent more than 12 h perceived worse overall well-being (β=−0.074) and domains of stability (β=−0.029), attachment (β=−0.019), and enjoyment (β=−0.025). Older age of parents predicted worse overall well-being (β=−0.003).

### Aim 3: differences between mothers and fathers

Regarding the comparison between fathers and mothers, there was no significant difference in most of the variables, except in the level of autism severity measured by the CABS (*p* = 0.028), time on caretaking of the children per day (*p* = 0.001), and efficacy subscale score of the PSOC (*p* = 0.029). Fathers reported a slightly higher level of autism severity than that reported by mothers (mean 16.9 [SD 4.8] vs. mean 15.0 [SD 5.0]). Notably, nearly half (47.9%) of the fathers spent fewer than 3 h on caretaking of the child per day, while more than half (57.2%) of the mothers spent more than 7 h on taking care of the child. Although no statistical difference was found in the total score of PSOC (*p* = 0.056) between mothers and fathers, fathers reported a higher score in the efficacy subscale of PSOC than that reported by mothers (mean 33.3[SD 8.2] vs. mean 30.8[SD 6.2]).

## Discussion

In this study, parents of autistic children reported lower capability well-being (mean: 0.672) compared to the general population in China (mean: 0.848) [34] and in several European countries (mean: 0.83–0.89) [30, 33, 37]. Although most were physically healthy, their well-being levels were similar to those of individuals with depression (mean: 0.63), and notably lower than those with other chronic conditions (mean: 0.79–0.85) [37]. These findings suggest that caring for an autistic child imposes challenges that can significantly impair parents’ capability well-being. Prior studies have showed that these parents often face elevated stress, fatigue, and sleep deprivation [22, 38, 39]. Limited social support, intensive caregiving responsibilities, and the high cost of therapies further restrict their abilities to pursue meaningful goals and activities [40–42]. In China, these challenges are compounded by a lack of well-developed diagnostic and intervention systems, leaving families with minimal formal support [7]. Cultural factors further intensify the burden: disability is often viewed as a source of family shame and a reflection of parental failure [8, 10], leading to social discrimination and internalized self-blame among parents [9, 10]. These structural and cultural challenges likely contribute to the low well-being scores observed in this population. Our findings underscore the urgent need to raise public awareness, reduce stigma, and expand support services for parents of autistic children in China. A more inclusive and compassionate society can play a vital role in improving their well-being and fostering a more supportive environment for affected families.

Despite the generally low levels of capability well-being among parents of autistic children, fathers reported slightly higher well-being than mothers, particularly in the domains of autonomy, achievement, and enjoyment. However, the effect size of these differences was small, suggesting that while the gender disparity is statistically significant, the practical significance is modest. Even after adjusting for child, parent, and family-level factors, mothers reported marginally lower well-being than fathers in the domains of autonomy and achievement. These findings are consistent with prior research showing that mothers of children with intellectual disabilities face a higher risk of poor mental health and well-being compared to fathers [13]. Caring for an autistic child often disrupts caregivers’ employment, with mothers more likely to reduce working hours or leave the workforce entirely, assuming the role of primary caregiver, while fathers typically remain in the workforce as the main breadwinner [14, 43]. Prior studies suggest that time spent outside the home, such as through paid employment, can protect parental well-being [9, 44, 45]. In our study, mothers reported spending significantly more time on daily caregiving than fathers, suggesting that fathers may be less involved in childcare. This unequal division of labor likely limits mothers’ opportunities to engage in social and recreational activities, diminishing their sense of autonomy and achievement. In China, traditional gender roles and cultural expectations place additional burdens on mothers, who are expected to manage both the child’s development and household responsibilities [11, 12]. Chinese mothers of autistic children are often not only caregivers but also act as therapeutic coaches for their children’s development, which intensifies their caregiving burden and further limits time for personal pursuits [46]. Additionally, gender differences in self-reported parenting efficacy may contribute to these well-being disparities. Fathers often express greater confidence in their parenting capabilities, possibly due to their limited involvement in daily caregiving, which may reduce their awareness of their children’s needs [47]. These findings underscore the importance of increasing fathers’ awareness and engagement in their children’s care. Promoting shared caregiving responsibilities and equipping fathers with appropriate resources may help bridge gender gaps in parental roles and improve overall family well-being.

Parenting sense of competence—measured in this study through parents’ satisfaction and self-efficacy in their parenting role—emerged as a key predictor of capability well-being. We included both PSOC and ICECAP-A in our analysis to examine how parenting competence relates to broader well-being. PSOC measures how capable and satisfied parents feel in their caregiving role, while ICECAP-A evaluates a more holistic view of well-being that extends beyond the caregiving role. Using both tools allows us to see whether feeling competent as a parent is linked to greater freedom and well-being in life. The results showed that higher scores on parenting competence were positively associated with the overall capability well-being score and each of its five domains. This aligns with previous research showing that stronger perceived parenting competence is linked to improved parental well-being and quality of life [26, 42]. Parents who feel more competent in their role may be more likely to experience positive functioning in daily life—and vice versa. Consistent with prior findings [48], higher socioeconomic status, reflected in education level and household income, was also associated with better capability well-being. Conversely, several risk factors were identified that may undermine overall well-being or specific domains. Notably, parents of children with more severe autism symptoms reported lower well-being, particularly in the domains of attachment and enjoyment. This may be due to the communication and behavioral difficulties that hinder secure, reciprocal parent–child relationships [26]. In contrast, parents of children currently receiving intervention reported better well-being in the domain of attachment. This could be attributed to improvements in the child’s symptoms or increased access to information and social support through intervention services. These findings underscore the need to explore which specific symptoms most strongly impact parental well-being. Additionally, parents who spent more hours caregiving reported lower well-being overall, as well as in the domains of stability, attachment, and enjoyment. Interestingly, prior research suggests that it may not be the number of hours alone, but the level of perceived time pressure, that affects mental health and well-being [23]. Future studies should examine parents’ subjective experiences of time pressure to better understand the psychological burden of caregiving.

While prior research has explored broader well-being constructs (e.g. stress, social isolation) in parents of autistic children, fewer studies have applied the capability approach to this population, particularly in the Chinese context. Unlike earlier studies that focused primarily on health-related problems or the negative impacts of parenting, our study emphasizes how positive aspects of parental well-being are affected, with particular attention to differences between mothers and fathers. Given the gender disparities observed in well-being and parenting experiences, tailored interventions are needed for mothers and fathers. For mothers, interventions could reduce their caregiving load by encouraging shared responsibilities and giving them more time for themselves. Examples include providing respite care or flexible work options to ease their stress and improve their sense of autonomy and achievement. For fathers, interventions should raise awareness about the importance of their involvement in caregiving. Programs could teach fathers about their child’s needs and how they can contribute more to daily tasks. Further research is needed to explore fathers’ willingness, capacity, and barriers to caregiving—information that could inform policy and service provision to better support paternal engagement. Additionally, psychosocial support services are especially needed for vulnerable subgroups, such as parents facing high caregiving demands or low socioeconomic status. Finally, since parenting competence emerged as a significant predictor of capability well-being, future prevention and intervention programs should consider it a key target for improving parental outcomes.

This study has several limitations. First, due to the lack of preference-based data in the Chinese population, we used UK tariffs to calculate ICECAP-A index scores, which may affect measurement validity. Second, since the data were self-reported, recall bias—particularly regarding caregiving time and household income—could be present. Third, the cross-sectional design limits causal inference, creating potential bidirectional relationships between predictors and capability well-being. Future longitudinal studies should track parental well-being over time to clarify causal links. Fourth, most participants were recruited from intervention programs or institutions, likely resulting in a sample biased toward those more actively engaged in services and with higher socioeconomic status than the general Chinese population. Fifth, since mothers and fathers came from different families, comparisons may be confounded by the fact that fathers cared for children with relatively more severe autism. Despite this, fathers reported better capability well-being than mothers, suggesting the gender disparity may be underestimated. Future research should assess both parents of the same child. Sixth, the small proportion of fathers and the broad age range of children (1–17 years) limit generalizability. Future studies should examine parental well-being across specific age groups. Additionally, the use of parent-reported measures to assess autism severity may introduce potential rater bias. Parents’ own emotional distress or caregiving burden may influence their perceptions and reports of their child’s behavioral difficulties, potentially leading to inflated severity ratings [49]. This bias is particularly relevant in studies of parental well-being, where parent and child variables are reported by the same individual. Future research could benefit from incorporating clinician assessments or multi-informant approaches to mitigate this risk. Lastly, other factors influencing parental well-being—such as social support, employment status, coping skills, and parenting styles—were not explored and warrant further investigation.

## Conclusions

Parents of autistic children reported an impaired capability well-being compared to that of general populations and patients with other chronic conditions. After controlling for other confounders, maternal scores of well-being were relatively lower than paternal scores in the domains of autonomy and achievement. Given that gender disparities on perception of well-being and parenting experiences, professionals must prioritize gender-specific intervention programs tailored to the distinct needs of mothers and fathers, and actively promote and facilitate father involvement in the childcare. Prevention and intervention programs must systematically target parenting competence as a core component of improving parental well-being. Evidence-based psychosocial support services need to be specifically designed for high-risk parent populations, especially those facing intensive caregiving burdens, socioeconomic disadvantages, or parenting autistic children with more severe symptoms.

## Data Availability

All data generated or analyzed during this study are included in this published article.

## References

[1] American Psychiatric Association, American Psychiatric Association. DSM-5 task force. Diagnostic and statistical manual of mental disorders: DSM-5. Washington, D.C.: American Psychiatric Association; 2013. https://catalog.lib.unc.edu/catalog/UNCb7450240.

[2] CDC. Data and Statistics on Autism Spectrum Disorder. Autism Spectrum Disorder (ASD). 2024. https://www.cdc.gov/autism/data-research/index.html. Accessed 18 June 2024.

[3] Grebe SC, Mire SS, Kim H, et al. Comparing fathers’ and mothers’ perspectives about their child’s autism spectrum disorder. J Autism Dev Disord. 2022;52:1841–54. 34027629 10.1007/s10803-021-05077-7

[4] Bauminger N, Solomon M, Rogers SJ. Externalizing and internalizing behaviors in ASD. Autism Res. 2010;3:101–12. 20575109 10.1002/aur.131 PMC5659193

[5] Vernhet C, Michelon C, Dellapiazza F, et al. Perceptions of parents of the impact of autism spectrum disorder on their quality of life and correlates: comparison between mothers and fathers. Qual Life Res. 2022;31:1499–508. 34822048 10.1007/s11136-021-03045-3

[6] Li Z, Niu X, Wong PCM, et al. Factors influencing timely diagnosis of autism in China: an application of andersen’s behavioral model of health services use. BMC Psychiatry. 2025;25:143. 39966821 10.1186/s12888-025-06590-0 PMC11837288

[7] Su X, Cai RY, Uljarević M. Predictors of mental health in Chinese parents of children with autism spectrum disorder (ASD). J Autism Dev Disord. 2018;48:1159–68. 29127642 10.1007/s10803-017-3364-1

[8] Mak WWS, Kwok YTY. Internalization of stigma for parents of children with autism spectrum disorder in Hong Kong. Soc Sci Med. 2010;70:2045–51. 20362379 10.1016/j.socscimed.2010.02.023

[9] Wang H, Hu X, Han ZR. Parental stress, involvement, and family quality of life in mothers and fathers of children with autism spectrum disorder in mainland China: a dyadic analysis. Res Dev Disabil. 2020;107:103791. 33091710 10.1016/j.ridd.2020.103791

[10] Wang H, Liu S, Xu J, et al. Daily experiences and well-being of Chinese parents of children with autism. Autism. 2023;27:1560–74. 36594108 10.1177/13623613221144191

[11] Hu X, Han ZR, Bai L, et al. The mediating role of parenting stress in the relations between parental emotion regulation and parenting behaviors in Chinese families of children with autism spectrum disorders: a dyadic analysis. J Autism Dev Disord. 2019;49:3983–98. 31197635 10.1007/s10803-019-04103-z PMC6751273

[12] Ng CSM, Fang Y, Wang Z, et al. Potential factors of parenting stress in Chinese parents of children with autism spectrum disorder: a systematic review. Focus Autism Other Dev Disabil. 2021;36:237–48.

[13] Dunn K, Kinnear D, Jahoda A, et al. Mental health and well-being of fathers of children with intellectual disabilities: systematic review and meta-analysis. BJPsych Open. 2019;5:e96. 31694727 10.1192/bjo.2019.75 PMC6854361

[14] Paynter J, Davies M, Beamish W. Recognising the forgotten man: fathers’ experiences in caring for a young child with autism spectrum disorder. J Intellect Dev Disabil. 2018;43:112–24.

[15] Ozturk Y, Riccadonna S, Venuti P. Parenting dimensions in mothers and fathers of children with autism spectrum disorders. Res Autism Spectr Disord. 2014;8:1295–306.

[16] Flippin M, Crais ER. The need for more effective father involvement in early autism intervention: a systematic review and recommendations. J Early Interv. 2011;33:24–50.

[17] Seymour M, Giallo R, Wood CE. The psychological and physical health of fathers of children with autism spectrum disorder compared to fathers of children with long-term disabilities and fathers of children without disabilities. Res Dev Disabil. 2017;69:8–17. 28778051 10.1016/j.ridd.2017.07.018

[18] Upton D, Upton P. Quality of life and Well-Being. In: Psychology of wounds and wound care in clinical practice. Cham: Springer International Publishing, 2015. pp. 85–111.

[19] Emmanuel CJ, Knafl KA, Hodges EA, et al. Family members’ experience of well-being as racial/ethnic minorities raising a child with a neurodevelopmental disorder: a qualitative meta‐synthesis. Res Nurs Health. 2022;45:314–26. 35141915 10.1002/nur.22217

[20] Karst JS, Van Hecke AV. Parent and family impact of autism spectrum disorders: a review and proposed model for intervention evaluation. Clin Child Fam Psychol Rev. 2012;15:247–77. 22869324 10.1007/s10567-012-0119-6

[21] Ten Hoopen LW, De Nijs PFA, Duvekot J, et al. Caring for children with an autism spectrum disorder: factors associating with health- and care-related quality of life of the caregivers. J Autism Dev Disord. 2022;52:4665–78. 34724164 10.1007/s10803-021-05336-7 PMC9556348

[22] Warreman EB, Lloyd SE, Nooteboom LA, et al. Psychological, behavioural, and physical aspects of caregiver strain in autism-caregivers: a cohort study. eClinicalMedicine. 2023;64:102211. 37767192 10.1016/j.eclinm.2023.102211 PMC10520302

[23] Sawyer MG, Bittman M, La Greca AM, et al. Time demands of caring for children with autism: what are the implications for maternal mental health? J Autism Dev Disord. 2010;40:620–8. 19949845 10.1007/s10803-009-0912-3

[24] Meleady J, Clyne C, Braham J, et al. Positive contributions among parents of children on the autism spectrum: a systematic review. Res Autism Spectr Disord. 2020;78:101635.

[25] Myers BJ, Mackintosh VH, Goin-Kochel RP. My greatest joy and my greatest heart ache: parents’ own words on how having a child in the autism spectrum has affected their lives and their families’ lives. Res Autism Spectr Disord. 2009;3:670–84.

[26] Mathew NE, Burton KLO, Schierbeek A, et al. Parenting preschoolers with autism: socioeconomic influences on wellbeing and sense of competence. World J Psychiatr. 2019;9:30–46. 30915270 10.5498/wjp.v9.i2.30 PMC6422881

[27] Sen A. Commodities and capabilities. New Delhi: Oxford University Press; 1987.

[28] Alkire S. Valuing freedoms. Oxford: Oxford University Press; 2002.

[29] Rencz F, Mitev AZ, Jenei B, et al. Measurement properties of the ICECAP-A capability well-being instrument among dermatological patients. Qual Life Res. 2022;31:903–15. 34370186 10.1007/s11136-021-02967-2 PMC8921030

[30] Al-Janabi H, Flynn N, Coast T. Development of a self-report measure of capability wellbeing for adults: the ICECAP-A. Qual Life Res. 2012;21:167–76. 21598064 10.1007/s11136-011-9927-2 PMC3254872

[31] Baji P, Farkas M, Dobos Á, et al. Capability of well-being: validation of the Hungarian version of the ICECAP-A and ICECAP-O questionnaires and population normative data. Qual Life Res. 2020;29:2863–74. 32468403 10.1007/s11136-020-02542-1 PMC7561558

[32] Zhang H, Chen S, Yu J, et al. Association between adherence to behavioral intervention and capability well-being among parents of autistic children: a cross-sectional study from China. BMC Psychiatry. 2024;24:922. 39696063 10.1186/s12888-024-06394-8 PMC11657610

[33] Flynn TN, Huynh E, Peters TJ, et al. Scoring the Icecap-a capability instrument. Estimation of a UK general population tariff. Health Econ. 2015;24:258–69. 24254584 10.1002/hec.3014 PMC4322472

[34] Tang C, Xiong Y, Wu H, et al. Adaptation and assessments of the Chinese version of the ICECAP-A measurement. Health Qual Life Outcomes. 2018;16:45. 29530092 10.1186/s12955-018-0865-3 PMC5848585

[35] Ngai F-W, Wai-Chi Chan S, Holroyd E. Translation and validation of a Chinese version of the parenting sense of competence scale in Chinese mothers. Nurs Res. 2007;56:348–54. 17846556 10.1097/01.NNR.0000289499.99542.94

[36] Sun X, Allison C, Auyeung B, et al. What is available for case identification in autism research in Mainland China? Res Autism Spectr Disord. 2013;7:579–90.

[37] Mitchell PM, Al-Janabi H, Richardson J, et al. The relative impacts of disease on health status and capability wellbeing: a multi-country study. PLoS ONE. 2015;10:e0143590. 26630131 10.1371/journal.pone.0143590 PMC4667875

[38] Giallo R, Wood CE, Jellett R, et al. Fatigue, wellbeing and parental self-efficacy in mothers of children with an autism spectrum disorder. Autism. 2013;17:465–80. 21788255 10.1177/1362361311416830

[39] Seymour M, Wood C, Giallo R, et al. Fatigue, stress and coping in mothers of children with an autism spectrum disorder. J Autism Dev Disord. 2013;43:1547–54. 23124359 10.1007/s10803-012-1701-y

[40] McCann D, Bull R, Winzenberg T. The daily patterns of time use for parents of children with complex needs: a systematic review. J Child Health Care. 2012;16:26–52. 22308543 10.1177/1367493511420186

[41] Sharpe DL, Baker DL. Financial issues associated with having a child with autism. J Fam Econ Issues. 2007;28:247–64.

[42] Vasilopoulou E, Nisbet J. The quality of life of parents of children with autism spectrum disorder: a systematic review. Res Autism Spectr Disord. 2016;23:36–49.

[43] Montes G, Halterman JS. Child care problems and employment among families with preschool-aged children with autism in the United States. Pediatrics. 2008;122:e202-8. 18595965 10.1542/peds.2007-3037

[44] Eisenhower A, Blacher J. Mothers of young adults with intellectual disability: multiple roles, ethnicity and well-being. J Intellect Disabil Res. 2006;50:905–16. 17100951 10.1111/j.1365-2788.2006.00913.x PMC3071694

[45] Olsson MB, Hwang CP. Well-being, involvement in paid work and division of child‐care in parents of children with intellectual disabilities in Sweden. J Intellect Disabil Res. 2006;50:963–9. 17100956 10.1111/j.1365-2788.2006.00930.x

[46] Zhou T, Yi C. Parenting styles and parents’ perspectives on how their own emotions affect the functioning of children with autism spectrum disorders. Fam Process. 2014;53:67–79. 24400727 10.1111/famp.12058

[47] Hartley SL, Schultz HM. Support needs of fathers and mothers of children and adolescents with autism spectrum disorder. J Autism Dev Disord. 2015;45:1636–48. 25433405 10.1007/s10803-014-2318-0 PMC4442745

[48] Turnage D, Conner N. Quality of life of parents of children with autism spectrum disorder: an integrative literature review. J Spec Pediatr Nurs. 2022;27:e12391. 35986656 10.1111/jspn.12391

[49] De Los Reyes A, Kazdin AE. Informant discrepancies in the assessment of childhood psychopathology: a critical review, theoretical framework, and recommendations for further study. Psychol Bull. 2005;131:483–509. 16060799 10.1037/0033-2909.131.4.483

